# Colorectal Cancer Screening Uptake Across Age Groups in an Academic Health System

**DOI:** 10.1016/j.amepre.2026.108365

**Published:** 2026-05-27

**Authors:** Patrick Casebolt, Kathleen Rice, Victoria Harris, Parag Agnihotri, Ming Tai-Seale, Lin Liu, Maria Elena Martinez, Samir Gupta, Joshua Demb

**Affiliations:** 1 American Cancer Society, Atlanta, Georgia; 2 Population Health Services Organization, University of California, San Diego Health System, San Diego, California; 3 Division of Geriatrics, University of California, San Diego, California; 4 Department of Family Medicine, University of California, San Diego, CA; 5 Division of Bioinformatics, Department of Medicine, University of California, San Diego, California; 6 Herbert Wertheim School of Public Health and Human Longevity Science, University of California, San Diego, California; 7 Division of Gastroenterology, University of California, San Diego, California; 8 Jennifer Moreno Veterans Affairs San Diego Healthcare System, San Diego, California; 9 Department of Radiation Medicine and Applied Sciences, University of California, San Diego, California

## Abstract

**Introduction:**

This study aimed to evaluate colorectal cancer screening uptake in an academic setting after updated guidelines to initiate screening at age 45 years, focusing on differences between adults aged 45–49 years and 50–75 years, and by sociodemographic characteristics.

**Methods:**

This was a retrospective cohort of insured, average risk adults aged 45–75 years receiving care in the University of California San Diego Health System between 2021 and 2023. Electronic health records were used to capture colorectal cancer screening uptake and modality, individual-level sociodemographic factors, and linkage to ZIP code-level social vulnerability measures. Colorectal cancer screening adherence was measured by calendar year and compared between ages 45–49 years and 50–75 years using multivariable Poisson regression with robust standard errors.

**Results:**

Adults aged 45–49 years experienced increased screening adherence from 20% in 2021 to 46% in 2023. Their screening adherence likelihood were lower than adults aged 50–75 years across all years (2021 adjusted prevalence ratio [aPR]=0.27, 95% CI=0.25, 0.29; 2022 aPR=0.45, 95% CI=0.43, 0.48; 2023 aPR=0.59, 95% CI= 0.56, 0.62). Colonoscopy was the primary screening modality, though adults aged 45–49 years increased stool-based testing from 3% in 2021 to 9% in 2023. All racial and ethnic groups showed increased screening adherence from 2021 to 2023, leaving existing screening disparities unchanged.

**Conclusions:**

Adults aged 45–49 years had lower colorectal cancer screening adherence likelihood than adults aged 50–75 years and had increasing interest in stool-based test modalities between 2021 and 2023. Despite concerns guideline changes could increase screening disparities, colorectal cancer screening adherence increased across all racial and ethnic groups, though gaps between groups did not change. The findings highlight screening promotion successes at one academic center, while emphasizing the need for focused interventions to continue minimizing disparities and improving adherence.

## INTRODUCTION

Colorectal cancer (CRC) is the fourth most common cancer and second leading cause of cancer death in the U.S.^1^ Increased incidence among adults aged 20–49 years has led to CRC being the leading cause of cancer death in adults aged <50 years.^1-3^ Epidemiologic trends point to a birth cohort effect, where adults born after 1960 have increased CRC risk compared with those born before 1960.^4^ In response to escalating birth cohort-related CRC incidence and mortality trends, organizations including the American Cancer Society and U.S. Preventive Services Task Force (USPSTF) updated screening guidelines to recommend starting screening at age 45 years instead of 50 years.^5-7^

Although modeling indicates starting screening at 45 years reduces incident and fatal CRC risk, there is limited evidence about whether adults aged 45–49 years are willing and able to undergo CRC screening.^8^ Data from after the 2021 USPSTF guideline update found CRC screening completion among adults aged 45–49 years reached only 13.9%.^9^ Further, expanding screening eligibility to adults aged 45–49 years increased the screening-eligible pool by 21 million adults, a 22% increase.^10^ Taken together, there are concerns that guideline changes could exacerbate age-based screening disparities with continued lower participation for younger versus older age individuals, minimizing the potential modeled screening benefit.^9^ The large increase in screening-eligible adults could also exacerbate existing race and ethnicity screening disparities.^10,11^ Understanding screening behaviors among newly age-eligible adults could help maximize these benefits, though few studies to date have examined the screening behaviors of adults aged 45–49 years and how they might differ from adults aged 50–75 years.

To fill this evidence gap, this study characterizes CRC screening uptake in an academic health system, with a focus on differences between adults aged 45–49 years and aged 50–75 years.

## METHODS

### Study Population

The study cohort included adults aged 45–75 years at average risk for CRC, receiving care at the University of California San Diego Health (UC San Diego Health) between 2021 and 2023. UC San Diego Health is one of the largest health systems in Southern California, serving San Diego and Imperial counties. Adults qualified as “receiving care” if they had a primary care visit in the past 3 years. Individuals with a history of total colectomy, inflammatory bowel disease-associated colitis, prior CRC, prior polyps, or who were uninsured were excluded. The decision to exclude uninsured individuals was driven by concerns about potential fragmentation or incompleteness of preventive screening and follow-up records in this population, their high likelihood of receiving care in urgent or emergent settings, and the fact that only 2% of uninsured patients have an established primary care provider. Data were queried in January 2024, and analyses were completed between January 2024 and September 2025. The study was approved by the institutional review board of UC San Diego (IRB #806196); informed consent was waived.

### Measures

Electronic health records were used to obtain CRC screening date and modality, CRC diagnosis date, latest primary care visit date each year, birth year, sex assigned at birth, race and ethnicity, insurance payor and plan, mailed fecal immunochemical test (FIT) order, and residence ZIP code. Linked ZIP code-level data included urban or rural status and the Social Vulnerability Index (SVI). CRC screening dates and modalities (e.g., colonoscopy, computed tomography [CT] colonography, FIT, multi target stool-DNA [mt-sDNA], sigmoidoscopy) during the study period were recorded based on Current Procedural Terminology codes and laboratory results. Age was measured yearly over the course of the follow-up period. To examine differences between newly eligible and previously eligible populations, adults aged 45–49 years and aged 50–75 years were compared, and age was stratified into 5-year age groupings. Self-reported race and ethnicity were classified as non-Hispanic (NH) American Indian or Alaska Native (AIAN), Native Hawaiian or Pacific Islander (NHPI), Hispanic, NH Asian, NH Black, NH White, or Other (included multiracial or not specified). Insurance payor and plan were consolidated to compare private insurance plans to public insurance plans. Mailed FIT outreach invitations, as part of a screening program led by UC San Diego Health Population Health Services Outreach (PHSO) were measured. Across the UC San Diego Health primary care clinics, beginning in 2021 and occurring 3–4 times per year through 2023, PHSO selected a primary care clinic and mailed a FIT kit to a random sampling of 900–2,000 individuals with an active primary care provider and active insurance, if they were not up-to-date with CRC screening.

SVI is a scale developed by the Centers for Disease Control and Agency for Toxic Substances and Disease Registry to measure ZIP code-level demographic and socioeconomic factors that could adversely affect communities if impacted by hazards or other stressors. SVI was derived using data from the 2017–2021 American Community Survey data linked to the EHR-extracted patient residential ZIP codes.^12^ SVI is comprised of 16 variables across 4 component themes, socioeconomic status, household traits, ethnic minority status, and housing type and transportation. A composite percentile score was calculated by collecting the sum of percentiles for variables within each theme and measuring the sum across the component themes, scoring from 0 (least vulnerable) to 1 (most vulnerable).

CRC screening adherence, being up to date with CRC screening in each calendar year, was the primary outcome of interest. For each modality, adherence aligned with the approach for quality reporting required for health insurers under the Healthcare Effectiveness Data and Information Set (HEDIS) program. This corresponded to either completion of any screening test within the calendar year, or colonoscopy within the last 10 years, sigmoidoscopy or CT colonography within the last 5 years, mt-sDNA test within the last 3 years or FIT within the last year. CRC screening modality among those that completed screening was documented as a secondary outcome. To increase likelihood of capturing screening-indicated tests, earliest CRC screening test was identified, prioritizing noncolonoscopy screening modalities to avoid capturing a follow-up colonoscopy as the initial screening test.

### Statistical Analysis

Study participants were characterized by individual-level and ZIP code-level sociodemographic covariates. Wilcoxon rank-sum tests and chi-square tests with a two-sided *p*-value of 0.05 were used to measure differences in continuous and categorical covariates, respectively, between adults aged 45–49 years and aged 50–75 years. Cross-sectional screening adherence (yes/no) and modality were calculated for each follow-up year. A *p* for trend for each age group over the follow-up period was calculated, and interaction between age group and study year was tested to examine change in CRC screening uptake over time. The average marginal change in screening proportion for adults aged 45–49 years and aged 50–75 years during follow-up was calculated. Given the common screening uptake outcome, univariable and multivariable Poisson regression analyses with robust standard errors measured associations between age group (aged 45–49 years and aged 50–75 years) and CRC screening completion adjusted for sociodemographic factors, yielding prevalence ratios (PR) and corresponding 95% CI. Interaction between age and other covariates was tested to examine sociodemographic differences in CRC screening uptake by age group. Sensitivity analyses were conducted excluding individuals who were ordered a mailed FIT kit by PHSO to isolate how usual care screening uptake differed by age. R software version 4.2.2 was used to perform statistical analyses.^13^

## RESULTS

There were 27,201 adults in 2021, 28,256 adults in 2022 and 29,716 adults in 2023 aged 45–75 years receiving care within UC San Diego Health (Table 1). The median age in each year was 61 years (Q1–Q3: 53–68 years for all 3 years), with 15% of individuals aged 45–49 years each year (3,934 adults in 2021; 4,186 adults in 2022; 4,540 adults in 2023). The population was majority female (56% for all 3 years) and NH White (63% in 2021; 62% in 2022–2023), with 15% identifying as NH Asian across all 3 years, 11% identifying as Hispanic in 2021 and 2022 and 12% identifying as Hispanic in 2023. Most individuals had private insurance (68% in 2021; 69% in 2022; 70% in 2023) and 64% lived in ZIP codes with an SVI score below the 50^th^ percentile in 2021–2023. Mailed FIT kits were sent to 771 adults in 2021 (3%), 2,944 adults in 2022 (10%), and 2,194 adults in 2023 (7%). When stratified by age group, adults aged 45–49 years included fewer NH White adults and had greater proportions of Hispanic and NH Asian adults than those aged 50–75 years (Appendix Table 1, available online). In addition, a higher proportion of adults aged 45–49 years had private insurance in each year compared with adults aged 50–75 years.

Among screening-eligible adults, there were 18,243 adults (69%) in 2021, 19,788 adults (78%) in 2022, and 21,627 adults (79%) in 2023 who completed CRC screening (Table 2). When comparing screening adherence by 5-year age groupings, adults aged 45–49 years (20% in 2021; 34% in 2022; 46% in 2023) and adults aged 50–54 years (60% in 2021; 61% in 2022; 66% in 2023) had markedly lower screening adherence rates than adults aged 55–75 years, who had a screening completion rate of ≥76% in all 3 years (Figure 1). Although colonoscopy was the primary screening modality utilized overall, use of FIT (aged 45–49 years: 3% in 2021; 9% in 2022; 9% in 2023; *p*-trend<0.01) increased in adults aged 45–49 years and mt-sDNA testing (aged 45–49 years: 3% in 2021; 8% in 2022; 9% in 2023; *p*-trend<0.01; aged 50–75 years: 3% in 2021; 4% in 2022; 5% in 2023, *p*-trend<0.01) increased in both groups (Table 2). All racial and ethnic groups showed increases in screening adherence rates from 2021 to 2023, regardless of age group (Appendix Figure 1, available online).

Screening completion proportions increased for adults aged 45–49 years (Marginal effect=25%; 95% CI=25%, 26%) and aged 50–75 years (Marginal effect=3%; 95% CI=3%, 3%) between 2021 and 2023 (Table 3). In Poisson regression analyses, adults aged 45–49 years had lower likelihood of screening adherence than adults aged 50–75 years in all 3 years (2021 adjusted PR [aPR]=0.27, 95% CI=0.25, 0.29; 2022 aPR=0.45, 95% CI=0.43, 0.48; 2023 aPR=0.59, 95% CI=0.56, 0.62). There were no changes in relative prevalence of screening uptake over time across racial and ethnic groups compared to non-Hispanic White adults (Appendix Table 2, available online). In sensitivity analyses excluding adults sent a mailed FIT kit, the study population (Appendix Table 3, available online) and Poisson regression findings were not qualitatively different (Appendix Table 4, available online). No significant interaction effect with age group was seen by sex, race and ethnicity, insurance type, or SVI composite score.

## DISCUSSION

In this study characterizing CRC screening uptake within an academic health system, there were notable differences in screening adherence by age. Adults aged 45–49 years showed lower screening adherence relative to those aged 50–75 years. Over the 3 years, however, adults aged 45–49 years made significant strides, increasing their screening adherence rate from 20% in 2021 to 46% by the end of 2023. These findings were robust to sensitivity analyses accounting for active screening outreach used by the institution. Further, earlier screening initiation was not associated with increased screening disparities by race or ethnicity. Taken together, the results highlight meaningful progress and opportunities for improvement in CRC screening at a large academic medical center, given the recent guideline recommendation for screening initiation at age 45 years.

Although CRC screening uptake increased over the study period in both age groups, it remained significantly lower among adults aged 45–49 years compared with those aged 50–75 years. This study is among the first to evaluate screening uptake rates among adults aged 45–49 years after the 2021 USPSTF guideline update. A recent study leveraging data from the National Health Interview Survey found increasing screening uptake rates among adults aged 45–49 years from 21% in 2019 to 34% in 2023.^14^ Another study of endoscopy clinics at a large California academic medical center found the screening uptake rate among adults aged 45–49 years was only 11.6% after the 2018 American Cancer Society recommendation, markedly lower than the 53.9% uptake observed in adults aged 50–54 years.^15^ The findings point to a gradual screening uptake increase among adults aged 45–49 years as they gain awareness of their eligibility and health care practice adapts to updated guidelines.

As adults aged 45–49 years are further integrated into the screening eligible population, adapting screening strategies to their preferences will be crucial. A recent study found patients as young as 40 years and adults aged ≥50 years preferred noninvasive screening modalities, particularly a mt-sDNA test (Cologuard).^16^ Although colonoscopy was the preferred screening modality used among all screening adherent adults in this study, adults aged 45–49 years had noted increases in FIT and mt-sDNA test uptake between 2021 and 2023. The findings align with the Star et al.^14^ study, which found stool-based testing increases from 1% in 2019 to 7% by 2023. Limited increases in noninvasive testing among adults aged 50–75 years could be attributed to more prevalent screening adherence with colonoscopy before 2021. As noninvasive tests continue to emerge and become more desirable, it is imperative that health care providers discuss these colonoscopy alternatives with their patients, which could help maximize screening uptake.^17,18^

The study results also found all racial and ethnic groups experiencing increases in screening adherence between 2021 and 2023. Despite concerns that lowering the CRC screening starting age could exacerbate screening-related disparities, findings of unchanged screening gaps across groups indicated otherwise. Notably, Hispanic and NH Asian adults reached screening adherence rates comparable to NH White adults in 2023 and nearing or exceeding the National Colorectal Cancer Roundtable benchmark of “80% screening in every community.”^19^ The findings among Hispanic adults contrast with multiple studies that found Hispanic adults had lower odds of screening completion compared to NH White adults.^20-22^ In addition, NH Black adults had the second highest screening adherence rates in 2021 of all racial and ethnic groups, which could additionally be attributed to guidance before 2021 from the American College of Gastroenterology and the U.S. Multi-Society Task Force that African American adults initiate screening at the age of 45 years.^23,24^ Although the AIAN, NHPI, and NH Black adults had lower screening adherence rates compared with NH White adults, the observed increases across all racial and ethnic groups suggest standardized, system-wide preventive care processes were applied uniformly within the health system.

Prior research has leveraged administrative claims, survey and electronic health records data to examine screening uptake in adults aged 45–49 years, compared with other age groups.^9,25-28^ Multiple studies have adopted a pre-post methodology to compare screening uptake before and after the USPSTF guideline update, finding marked absolute and relative increases in screening uptake^9,25^ and provider orders for screening.^26^ This study within a single academic health system builds upon this foundation by further characterizing how screening behavior evolves within the health system in the 3 years immediately after the USPSTF guideline change. The sustained increases in screening rates between 2021 and 2023 likely reflect UC San Diego Health’s clinic-based usual care efforts to promote CRC screening adherence, including provider clinical reminders, patient-directed care gap outreach, and performance auditing with feedback to individual clinics and clinicians. Additional efforts, including development of a robust mailed FIT outreach program, have not been thoroughly assessed for their potential benefit overall and among this population of newly eligible adults. Although sensitivity analyses excluding individuals who received mailed FIT did not materially change study findings, a more rigorous comparison of screening uptake among invited individuals and sociodemographically comparable noninvited screening-eligible adults would provide further insight.

These study findings also establish a framework for future research. More evidence is needed regarding patient awareness of screening eligibility and access, as well as provider engagement and practice patterns. The slight increase in noninvasive test uptake may represent an opportunity to reach less health care-engaged adults or those who prefer colonoscopy alternatives. Further, stagnant uptake among adults aged 50–54 years could represent a broader concern necessitating strategic intervention among younger screening-eligible adults, not just those newly eligible. Future research should consider these trends to help prioritize identifying and evaluating targeted interventions to optimize screening uptake.

### Limitations

Key strengths include a large, demographically and socioeconomically diverse, average-risk screening population, reflective of the broader San Diego region. By including multiple screening modalities, the study more closely aligns with real-world implementation of CRC screening, where institutions must provide choices to establish screening touchpoints and ensure long-term adherence to screening.

Despite these strengths, this study has notable limitations. Only 1 institution was investigated, impacting the generalizability of the findings across diverse health systems. Restricting the analytic cohort to adults with recent primary care engagement could underrepresent individuals with inconsistent health care access. Exclusion of uninsured individuals, though necessary to appropriately measure guideline-eligible populations with access to preventive screening and follow-up care, limits generalizability of our findings to all at-risk adults, particularly those disproportionately impacted by lower CRC screening uptake and resultant increased CRC risk. The USPSTF guideline took effect in May 2021, meaning that adults aged 45–49 years lacked 3 full years of data to examine CRC screening uptake compared with other age groups. As this study did not measure the predictive impact of non-CRC-related preventive care, future studies should examine if health care maintenance tasks, like vaccinations and other routine screenings, impact CRC screening adherence or offer opportunities for cooperative promotion. Though ZIP code-level social vulnerability was adjusted for in the analysis, the distribution of individuals within various ZIP codes limited the ability to adjust for potential clustering effects. This study also did not adjust for COVID-19 disruptions, which likely contributed to lower overall screening rates in early 2021. Observed increases in screening uptake may reflect unmeasured lifestyle, health status, or health system practice patterns, unavailable within structured EHR data. Future studies comparing multiple health systems or with resources for manual chart abstraction may be better positioned to disentangle the role of these factors.

## CONCLUSIONS

Within UC San Diego Health, despite overall increasing screening adherence rates between 2021 and 2023, adults aged 45–49 years had persisting lower likelihood of screening completion compared with adults aged 50–75 years. Adults aged 45–49 years showed increasing preference for stool-based tests during the study period, highlighting a potential opportunity to further optimize screening uptake in this group. Across all racial and ethnic groups, screening uptake increased from 2021 through 2023, though screening uptake gaps remained unchanged. These findings reflect the typical care experience within a single academic health system and highlight areas where additional improvements could be made through focused interventions to ensure this group of younger age adults is most effectively integrated into the CRC screening eligible population.

## Supplementary Material

Supplementary Materials

Supplemental materials associated with this article can be found in the online version at https://doi.org/10.1016/j.amepre.2026.108365.

## Figures and Tables

**Figure 1.**
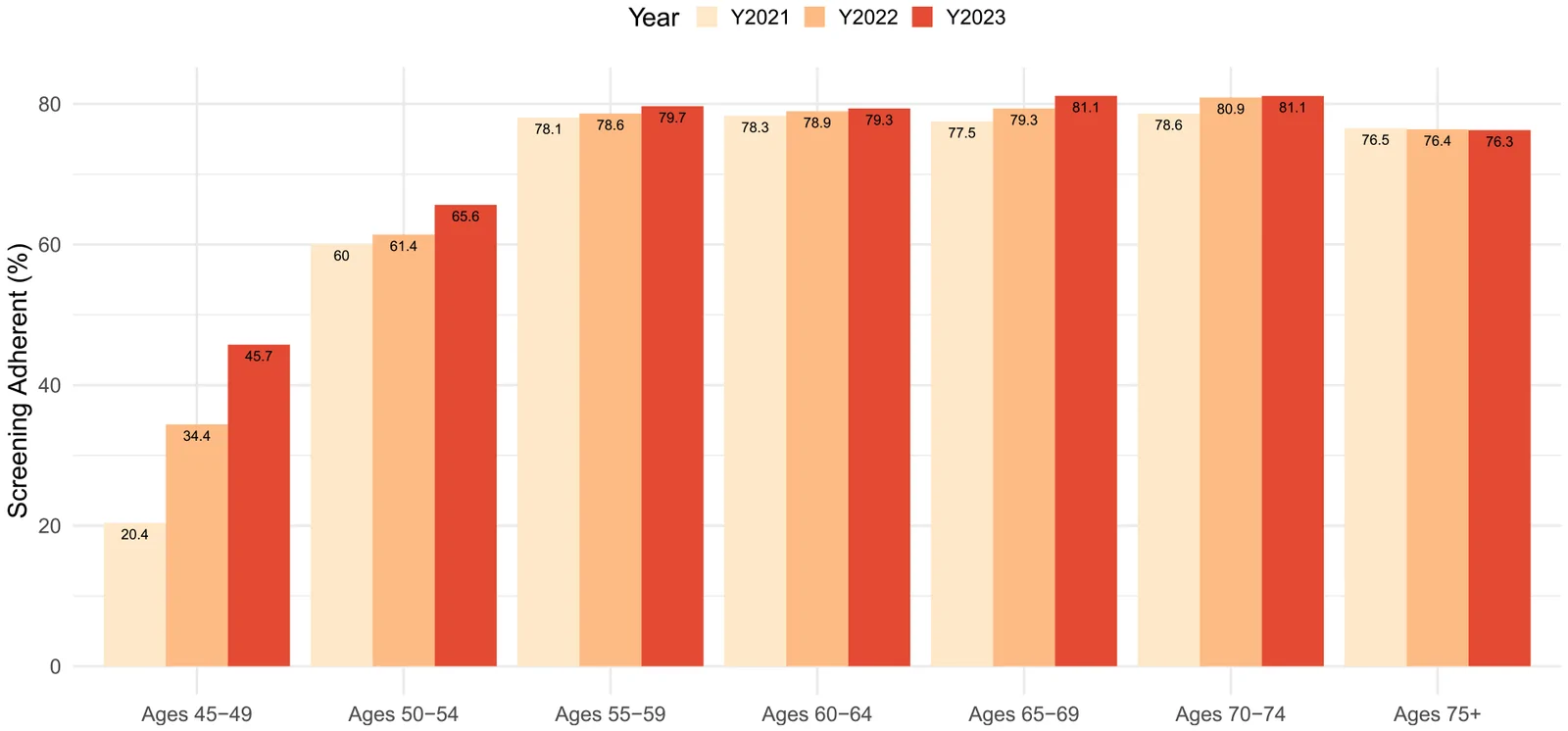
CRC screening adherence by year, stratified by age group. CRC, colorectal cancer.

**Table 1.** Descriptive Characteristics of Screening Eligible Adults Receiving Care at UC San Diego Health, Stratified by Year

Characteristic	2021 N=27,201	2022 N=28,256	2023 N=29,716
Age, median [Q1–Q3]	61.0 [53.0–68.0]	61.0 [53.0–68.0]	61.0 [53.0–68.0]
45–49	3,934 (14.5%)	4,186 (14.8%)	4,540 (15.3%)
50–54	3,977 (14.6%)	4,158 (14.7%)	4,348 (14.6%)
55–59	4,342 (16.0%)	4,321 (15.3%)	4,480 (15.1%)
60–64	4,389 (16.1%)	4.541 (16.1%)	4,821 (16.2%)
65–69	5,086 (18.7%)	5,241 (18.5%)	5,388 (18.1%)
70–74	4,664 (17.1%)	4,810 (17.0%)	5,103 (17.2%)
75+	809 (3.0%)	999 (3.5%)	1,036 (3.5%)
Sex, *n* (%)			
Male	11,898 (43.7%)	12,348 (43.7%)	12.952 (43.6%)
Female	15,303 (56.3%)	15,908 (56.3%)	16,764 (56.4%)
Race/ethnicity, *n* (%)			
AIAN	85 (0.3%)	86 (0.3%)	97 (0.3%)
Hispanic	2,974 (10.9%)	3,179 (11.3%)	3,498 (11.8%)
NH Asian	4,112 (15.1%)	4,280 (15.1%)	4,486 (15.1%)
NH Black	973 (3.6%)	1,023 (3.6%)	1,049 (3.5%)
NH White	17,095 (62.8%)	17,541 (62.1%)	18,266 (61.5%)
NHPI	130 (0.48%)	142 (0.5%)	147 (0.49%)
Other	1,832 (6.74%)	2,005 (7.1%)	2,173 (7.3%)
Insurance type, *n* (%)			
Private	18,404 (67.7%)	19,592 (69.3%)	20,815 (70.0%)
Public	8,308 (30.5%)	8,141 (28.8%)	8,166 (27.5%)
Unknown	489 (1.8%)	523 (1.9%)	735 (2.5%)
SVI Composite, median [Q1–Q3]	0.39 [0.24–0.60]	0.39 [0.24–0.60]	0.39 [0.24–0.60]
Q1	7,398 (27.2%)	7,684 (27.2%)	8,183 (27.5%)
Q2	9,899 (36.4%)	10,318 (36.5%)	10,741 (36.1%)
Q3	6,066 (22.3%)	6,239 (22.1%)	6,568 (22.1%)
Q4	3,838 (14.1%)	4,015 (14.2%)	4,224 (14.2%)
Mailed FIT receipt, *n* (%)	771 (2.8%)	2,944 (10.4%)	2,194 (7.4%)

AIAN, American Indian or Alaska Native; FIT, fecal immunochemical test; NH, non-Hispanic; NHPI, Native Hawaiian or Pacific Islander; Q, quartile; SVI, Social Vulnerability Index.

**Table 2.** Screening Completion and CRC Screening Modality Used Among Completers, Stratified by Age and Year

Screening modality	Age 45–49				Age ≥50			
	2021 N=3,934	2022 N=4,186	2023 N=4,540	*p*-trend	2021 N=23,267	2022 N=24,070	2023 N=25,176	*p*-trend
Unscreened, *n* (%)	3,132 (79.6%)	2,746 (65.6%)	2,463 (54.3%)	<0.01	5,826 (25.0%)	5,722 (23.8%)	5,626 (22.3%)	<0.01
Screened Overall, *n* (%)	802 (20.4%)	1,440 (34.4%)	2,077 (45.7%)		17,441 (75.0%)	18,348 (76.2%)	19,550 (77.7%)	
Colonoscopy	737 (91.9%)	1,180 (81.9%)	1,696 (81.7%)	<0.01	16,364 (93.8%)	16,859 (91.9%)	17,846 (91.3%)	0.46
CT Colonography	2 (0.3%)	0 (0.0%)	0 (0.0%)	0.99	2 (0.0%)	5 (0.0%)	6 (0.0%)	0.22
FIT	25 (3.1%)	129 (9.0%)	188 (9.0%)	<0.01	498 (2.9%)	663 (3.6%)	651 (3.3%)	<0.01
FIT-DNA	27 (3.4%)	120 (8.3%)	179 (8.6%)	<0.01	491 (2.8%)	738 (4.0%)	955 (4.9%)	<0.01
Sigmoidoscopy	11 (1.4%)	11 (0.8%)	14 (0.7%)	0.80	86 (0.5%)	83 (0.5%)	92 (0.5%)	0.95

*Note*: *p*-trend calculated via Wald test corresponding to trend in each modality within each age group over 3-year follow-up period.

CRC, colorectal cancer; CT, computed tomography; FIT, fecal immunochemical test.

**Table 3.** Absolute Screening Completion Rates and Poisson Regression Results for Screening Completion Prevalence Ratio by Age Group

Year	Age group, years	Screening completion proportion, (95% CI)	Unadjusted PR (95% CI)	Adjusted PR (95% CI)^a^
**Year 2021**	Age 45–49	20% (19%, 22%)	**0.27 (0.25, 0.29)**	**0.27 (0.25, 0.29)**
	Age ≥50	75% (74%, 76%)	Ref	Ref
**Year 2022**	Age 45–49	34% (33%, 36%)	**0.45 (0.43, 0.48)**	**0.45 (0.43, 0.48)**
	Age ≥50	76% (75%, 77%)	Ref	Ref
**Year 2023**	Age 45–49	46% (44%, 48%)	**0.59 (0.56, 0.62)**	**0.59 (0.56, 0.62)**
	Age ≥50	78% (77%, 79%)	Ref	Ref
**Average marginal effect^b^**	Age 45–49	**+25% (25%, 26%)**	—	—
	Age ≥50	**+3% (3%, 3%)**	—	—

a Adjusted for sex, race/ethnicity, insurance type, mailed FIT receipt, and SVI composite score.

b *p*-trend for both ages 45–49 and ≥50 over follow-up period was *p*<0.01.
