# Supplementary material for: Colorectal Cancer Screening Uptake Across Age Groups in an Academic Health System
Source: Am J Prev Med. Author manuscript; Available in PMC 2026 Nov 15. (PMC13770300; doi:10.1016/j.amepre.2026.108365)
Supplement: Supplementary file 1 [file NIHMS2222160-supplement-Supplementary_Materials.docx]

**APPENDIX**

**Appendix Figure 1**: CRC screening adherence by year, stratified by age and race and ethnic group.

**Appendix Table 1.** Descriptive characteristics of screening eligible adults receiving care at UC-San Diego Health, stratified by age group and year.

|  | **Year 2021** | | **Year 2022** | | **Year 2023** | |
| --- | --- | --- | --- | --- | --- | --- |
|  | **Ages 45-49** | **Ages ≥50** | **Ages 45-49** | **Ages ≥50** | **Ages 45-49** | **Ages ≥50** |
|  | ***N=3934*** | ***N=23267*** | ***N=4186*** | ***N=24070*** | ***N=4540*** | ***N=25176*** |
| Sex: |  |  |  |  |  |  |
| Male | 1700 (43.2%) | 10198 (43.8%) | 1805 (43.1%) | 10543 (43.8%) | 1935 (42.6%) | 11017 (43.8%) |
| Female | 2234 (56.8%) | 13069 (56.2%) | 2381 (56.9%) | 13527 (56.2%) | 2605 (57.4%) | 14159 (56.2%) |
| Race/Ethnicity: |  |  |  |  |  |  |
| AIAN | 25 (0.6%) | 60 (0.3%) | 22 (0.53%) | 64 (0.3%) | 16 (0.4%) | 81 (0.3%) |
| Hispanic | 587 (14.9%) | 2387 (10.3%) | 615 (14.7%) | 2564 (10.7%) | 672 (14.8%) | 2826 (11.2%) |
| NH Asian | 710 (18.0%) | 3402 (14.6%) | 744 (17.8%) | 3536 (14.7%) | 778 (17.1%) | 3708 (14.7%) |
| NH Black | 145 (3.7%) | 828 (3.6%) | 155 (3.7%) | 868 (3.6%) | 154 (3.4%) | 895 (3.6%) |
| NH White | 2076 (52.8%) | 15019 (64.6%) | 2219 (53.0%) | 15322 (63.7%) | 2448 (53.9%) | 15818 (62.8%) |
| NHPI | 28 (0.7%) | 102 (0.4%) | 29 (0.7%) | 113 (0.5%) | 32 (0.7%) | 115 (0.5%) |
| Other | 363 (9.2%) | 1469 (6.3%) | 402 (9.6%) | 1603 (6.7%) | 440 (9.7%) | 1733 (6.9%) |
| Insurance Type: |  |  |  |  |  |  |
| Private | 3391 (86.2%) | 15013 (64.5%) | 3617 (86.4%) | 15975 (66.4%) | 3941 (86.8%) | 16874 (67.0%) |
| Public | 440 (11.2%) | 7868 (33.8%) | 462 (11.0%) | 7679 (31.9%) | 479 (10.6%) | 7687 (30.5%) |
| Unknown | 103 (2.6%) | 386 (1.7%) | 107 (2.6%) | 416 (1.73%) | 120 (2.6%) | 615 (2.4%) |
| SVI Composite, median [Q1-Q3] | 0.39 [0.23-0.59] | 0.39 [0.24-0.60] | 0.39 [0.23-0.60] | 0.39 [0.24-0.60] | 0.39 [0.23-0.60] | 0.39 [0.24-0.60] |
| Q1 | 1127 (28.6%) | 6271 (27.0%) | 1159 (27.7%) | 6525 (27.1%) | 1267 (27.9%) | 6916 (27.5%) |
| Q2 | 1490 (37.9%) | 8409 (36.1%) | 1607 (38.4%) | 8711 (36.2%) | 1742 (38.4%) | 8999 (35.7%) |
| Q3 | 758 (19.3%) | 5308 (22.8%) | 814 (19.4%) | 5425 (22.5%) | 857 (18.9%) | 5711 (22.7%) |
| Q4 | 559 (14.2%) | 3279 (14.1%) | 606 (14.5%) | 3409 (14.2%) | 674 (14.8%) | 3550 (14.1%) |

**Appendix Table 2.** Poisson regression results for screening completion prevalence ratios by race and ethnicity.

|  |  | **Unscreened,**  **n (%)** | **Screened,** | **Adjusted** |
| --- | --- | --- | --- | --- |
|  |  |  | **n (%)** | **PR (95% CI)** |
| **Year 2021** | AIAN | 45 (41%) | 50 (59%) | 0.99 (0.74, 1.30) |
|  | Hispanic | 1172 (39%) | 1802 (61%) | 0.94 (0.89, 0.99) |
|  | NH Asian | 1404 (34%) | 2708 (66%) | 0.98 (0.94, 1.03) |
|  | NH Black | 332 (34%) | 641 (66%) | 0.98 (0.91, 1.07) |
|  | NHPI | 54 (42%) | 76 (59%) | 0.91 (0.72, 1.13) |
|  | Other | 776 (42%) | 1056 (58%) | 0.88 (0.83, 0.94) |
|  | NH White | 5185 (30%) | 11910 (70%) | Ref. |
| **Year 2022** | AIAN | 33 (38%) | 53 (62%) | 0.93 (0.70, 1.21) |
|  | Hispanic | 1107 (35%) | 2072 (65%) | 0.95 (0.91, 0.99) |
|  | NH Asian | 1296 (30%) | 2984 (70%) | 0.99 (0.95, 1.03) |
|  | NH Black | 332 (33%) | 691 (68%) | 0.96 (0.89, 1.04) |
|  | NHPI | 57 (40%) | 85 (60%) | 0.87 (0.70, 1.07) |
|  | Other | 797 (40%) | 1208 (60%) | 0.87 (0.82, 0.92) |
|  | NH White | 4846 (28%) | 12695 (72%) | Ref. |
| **Year 2023** | AIAN | 36 (37%) | 61 (63%) | 0.86 (0.66, 1.10) |
|  | Hispanic | 1107 (32%) | 2391 (68%) | 0.95 (0.90, 0.99) |
|  | NH Asian | 1215 (27%) | 3271 (73%) | 0.99 (0.95, 1.03) |
|  | NH Black | 327 (31%) | 722 (69%) | 0.94 (0.87, 1.01) |
|  | NHPI | 54 (37%) | 93 (63%) | 0.88 (0.71, 1.07) |
|  | Other | 762 (35%) | 1411 (65%) | 0.89 (0.84, 0.94) |
|  | NH White | 4588 (25%) | 13678 (75%) | Ref. |

***p-trend per group:** AIAN: 0.97; Hispanic: 0.16; NH Asian: 0.31; NH Black: 0.61; NHPI: 0.96; Other: 0.25

**Appendix Table 3.** Descriptive characteristics of screening eligible adults receiving care at UC-San Diego Health excluding those receiving mailed FIT outreach, stratified by year.

|  | **Y2021** | **Y2022** | **Y2023** |
| --- | --- | --- | --- |
|  | ***N=26430*** | ***N=25312*** | ***N=27522*** |
| Age | 61.0 [53.0-68.0] | 61.0 [53.0-68.0] | 61.0 [53.0-68.0] |
| Ages 45-49 | 3934 (14.9%) | 3392 (13.4%) | 4102 (14.9%) |
| Ages 50-54 | 3666 (13.9%) | 3793 (15.0%) | 3898 (14.2%) |
| Ages 55-59 | 4156 (15.7%) | 4055 (16.0%) | 4238 (15.4%) |
| Ages 60-64 | 4230 (16.0%) | 4280 (16.9%) | 4545 (16.5%) |
| Ages 65-69 | 4990 (18.9%) | 4695 (18.5%) | 5016 (18.2%) |
| Ages 70-74 | 4645 (17.6%) | 4263 (16.8%) | 4735 (17.2%) |
| Ages 75+ | 809 (3.1%) | 834 (3.3%) | 988 (3.6%) |
| Sex: |  |  |  |
| Male | 11557 (43.7%) | 11061 (43.7%) | 12043 (43.8%) |
| Female | 14873 (56.3%) | 14251 (56.3%) | 15479 (56.2%) |
| Race/Ethnicity: |  |  |  |
| AIAN | 82 (0.3%) | 72 (0.3%) | 84 (0.3%) |
| Hispanic | 2877 (10.9%) | 2801 (11.1%) | 3197 (11.6%) |
| NH Asian | 4001 (15.1%) | 3860 (15.2%) | 4176 (15.2%) |
| NH Black | 940 (3.6%) | 916 (3.6%) | 964 (3.5%) |
| NH White | 16632 (62.9%) | 15767 (62.3%) | 16981 (61.7%) |
| NHPI | 125 (0.5%) | 120 (0.5%) | 127 (0.5%) |
| Other | 1773 (6.7%) | 1776 (7.0%) | 1993 (7.2%) |
| Insurance Type: |  |  |  |
| Private | 17721 (67.0%) | 17619 (69.6%) | 19334 (70.2%) |
| Public | 8271 (31.3%) | 7249 (28.6%) | 7499 (27.2%) |
| Unknown | 438 (1.7%) | 444 (1.8%) | 689 (2.5%) |
| SVI Composite, median [Q1-Q3] | 0.39 [0.24-0.60] | 0.39 [0.24-0.60] | 0.39 [0.23-0.60] |
| Q1 | 7201 (27.2%) | 6986 (27.6%) | 7692 (27.9%) |
| Q2 | 9622 (36.4%) | 9260 (36.6%) | 9913 (36.0%) |
| Q3 | 5891 (22.3%) | 5538 (21.9%) | 6058 (22.0%) |
| Q4 | 3716 (14.1%) | 3528 (13.9%) | 3859 (14.0%) |

**Appendix Table 4.** Absolute screening completion rates and Poisson regression results for screening completion prevalence ratio by age group, excluding those receiving mailed FIT outreach.

|  |  | **Screening Completion**  **Proportion, (95% CI)** | **Unadjusted**  **PR (95% CI)** | **Adjusted**  **PR (95% CI)*** |
| --- | --- | --- | --- | --- |
| **Year 2021** | Ages 45-49 | 20% (19%, 22%) | 0.27 (0.25, 0.29) | 0.27 (0.25, 0.29) |
|  | Ages ≥50 | 76% (75%, 77%) | Ref. | Ref. |
| **Year 2022** | Ages 45-49 | 40% (38%, 42%) | 0.49 (0.46, 0.52) | 0.50 (0.47, 0.52) |
|  | Ages ≥50 | 81% (80%, 82%) | Ref. | Ref. |
| **Year 2023** | Ages 45-49 | 47% (45%, 49%) | 0.58 (0.55, 0.61) | 0.58 (0.56, 0.61) |
|  | Ages ≥50 | 81% (80%, 82%) | Ref. | Ref. |
| **Average**  **Marginal Effect** | Ages 45-49 | 26% (26%, 27%) | -- | -- |
|  | Ages ≥50 | 5% (5%, 5%) | -- | -- |

*Adjusted for sex, race/ethnicity, insurance type, and SVI composite score

**P-trend for both ages 45-49 and ages ≥50 over follow-up period was p<0.01
